# Successful treatment of severe electrolyte imbalance-induced cardiac arrest caused by adrenal tuberculosis with ECMO in the ED

**DOI:** 10.1186/s12245-021-00382-5

**Published:** 2021-09-20

**Authors:** Ning Yang, Liping Zhou, Xiaoye Mo, Guoqing Huang, Ping Wu

**Affiliations:** Department of Emergency Medicine, Xiangya Hospital, Central South University, 87 Xiangya Road, Changsha, 410008 Hunan China

**Keywords:** Cardiac arrest, Adrenal tuberculosis, Extracorporeal membrane oxygenation

## Abstract

**Background:**

Tuberculosis (TB) is a chronic infectious disease, common in China. TB bacteria can invade multiple organs throughout the body, but they rarely cause critical illness. We present a complex critically ill case in this report.

**Case presentation:**

A 40-year-old man suffered sudden cardiac arrest during an emergency room visit. Spontaneous circulation resumed after emergency cardiopulmonary resuscitation (CPR), but recurrent ventricular fibrillation and refractory cardiac shock emerged. Thereafter, extracorporeal membrane oxygenation (ECMO) was implemented to maintain hemodynamic stability. Blood test results revealed that the patient had severe electrolyte imbalance and adrenal insufficiency. Further imaging examination showed multiple tuberculosis lesions throughout the body, including the lungs, adrenal glands, and lumbar spine. In the end, the patient was successfully moved from the ICU after weaning from ECMO and the ventilator, and then transferred to an infectious disease specialist hospital for standard anti-tuberculosis therapy.

**Conclusions:**

ECMO has won the opportunity for the diagnosis and treatment of this young patient who suffered from a rare cause of cardiac arrest and finally achieved a good prognosis.

## Background

Patients with various causes of cardiac arrest (CA) are often encountered in the emergency department, and the success rate of treatment for CA is not very high, especially the success rate of cerebral resuscitation is not high. The case report presents a complex critically ill case. A patient with CA caused by rare cause has been successfully treated with severe technology such as extracorporeal life support.

## Case presentation

A 40-year-old man presented to the emergency department (ED) of the local hospital at approximately 11 am due to severe nausea, vomiting, and fatigue. He suddenly fell to the ground and lost consciousness while waiting. The on-site medical staff immediately determined cardiac arrest and performed CPR, defibrillation, and intubation. The patient’s spontaneous circulation resumed after approximately 4 min, but he had recurrent ventricular fibrillation and low blood pressure which required high doses of vasoactive drugs (norepinephrine 4μg/kg/min, terlipressin 1 mg iv q4h). His vital signs were not stable and his condition deteriorated because of increased blood lactate (3.2 mmol/l↑) and poor tissue perfusion. The ECMO team was urgently called to the ED to initiate VA-ECMO for the patient, after which he was transferred to the emergency ICU of a tertiary comprehensive hospital.

**Fig. 1:**
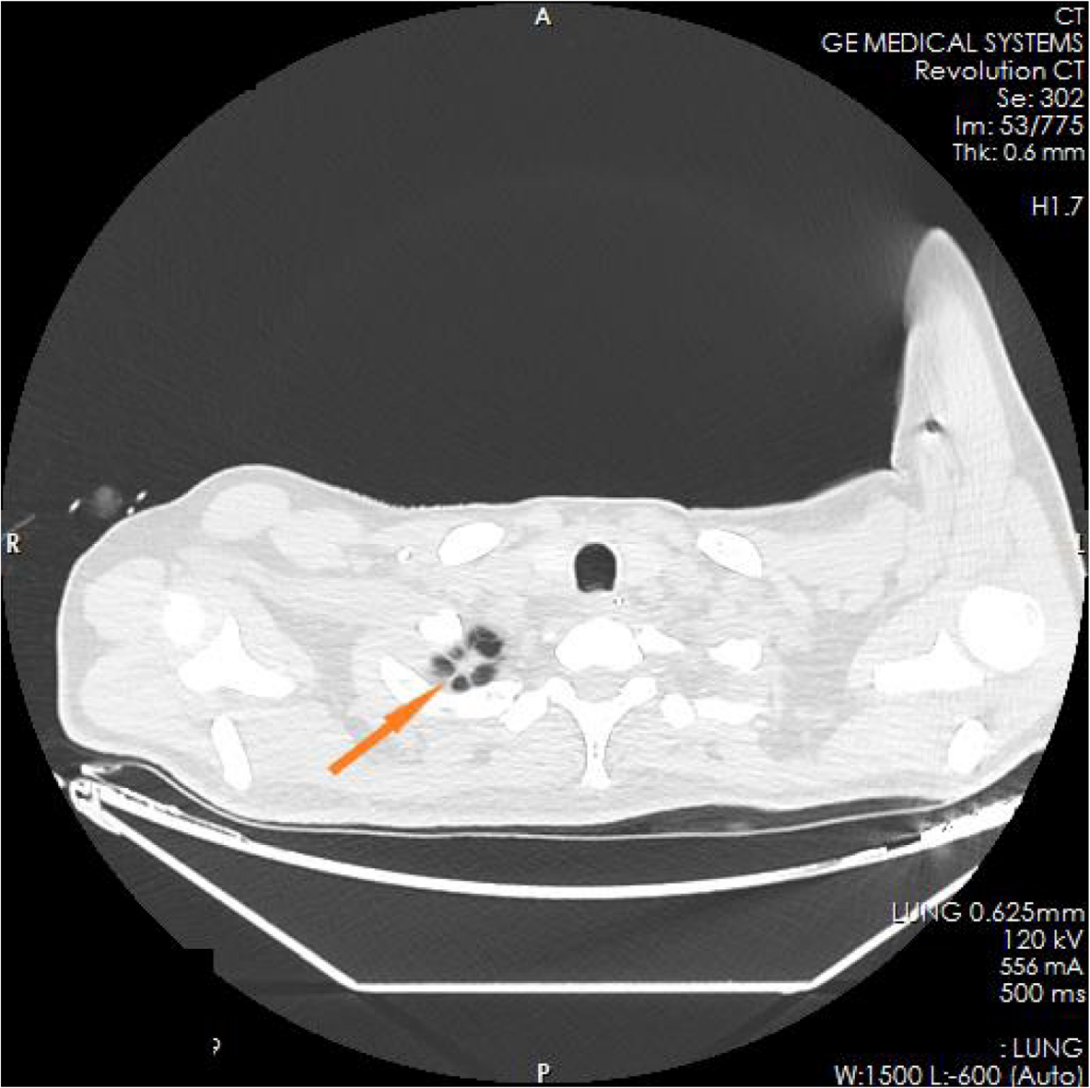
CT pictures displaying multiple tuberculosis lesions throughout the body

**Fig. 2:**
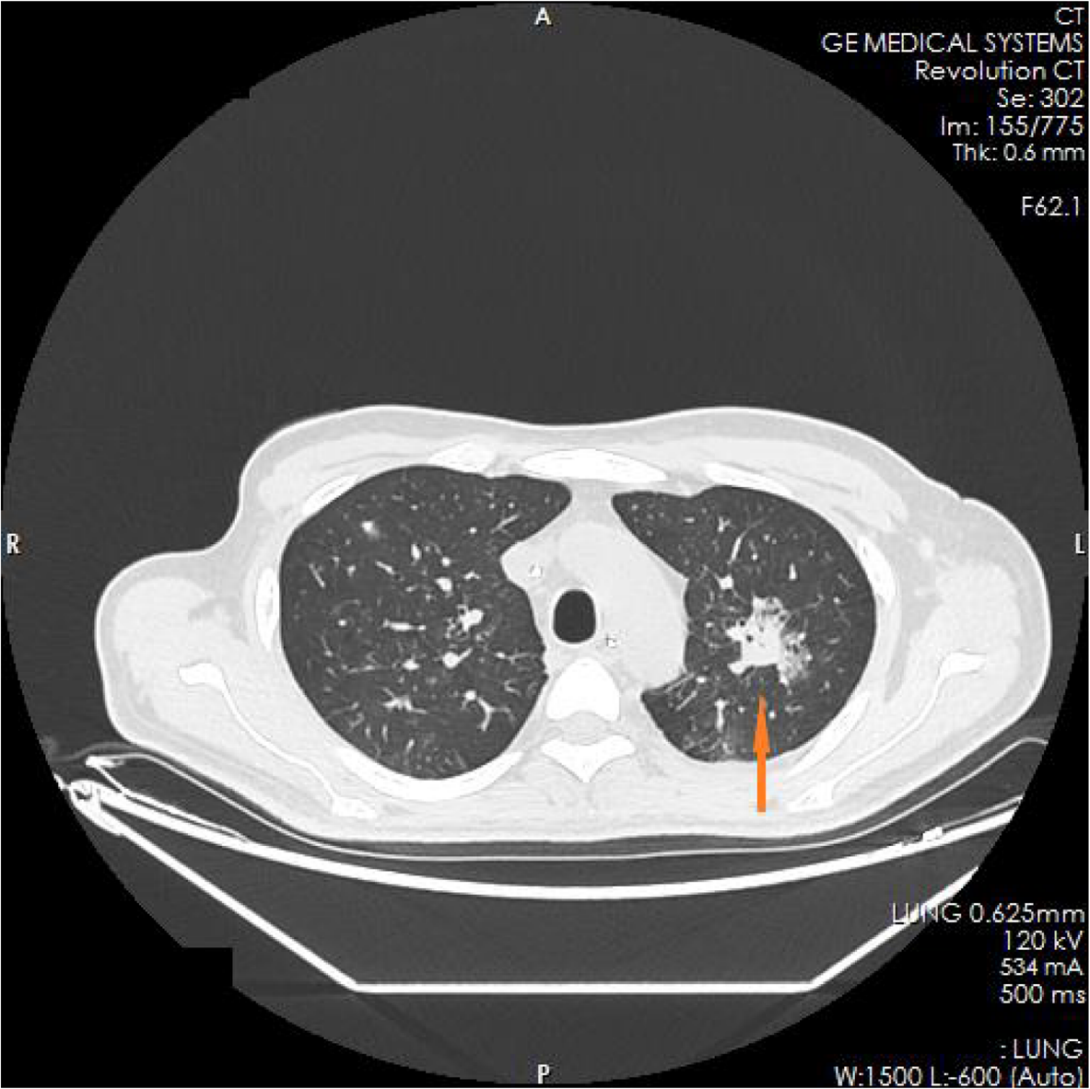
CT pictures displaying multiple tuberculosis lesions throughout the body

**Fig. 3:**
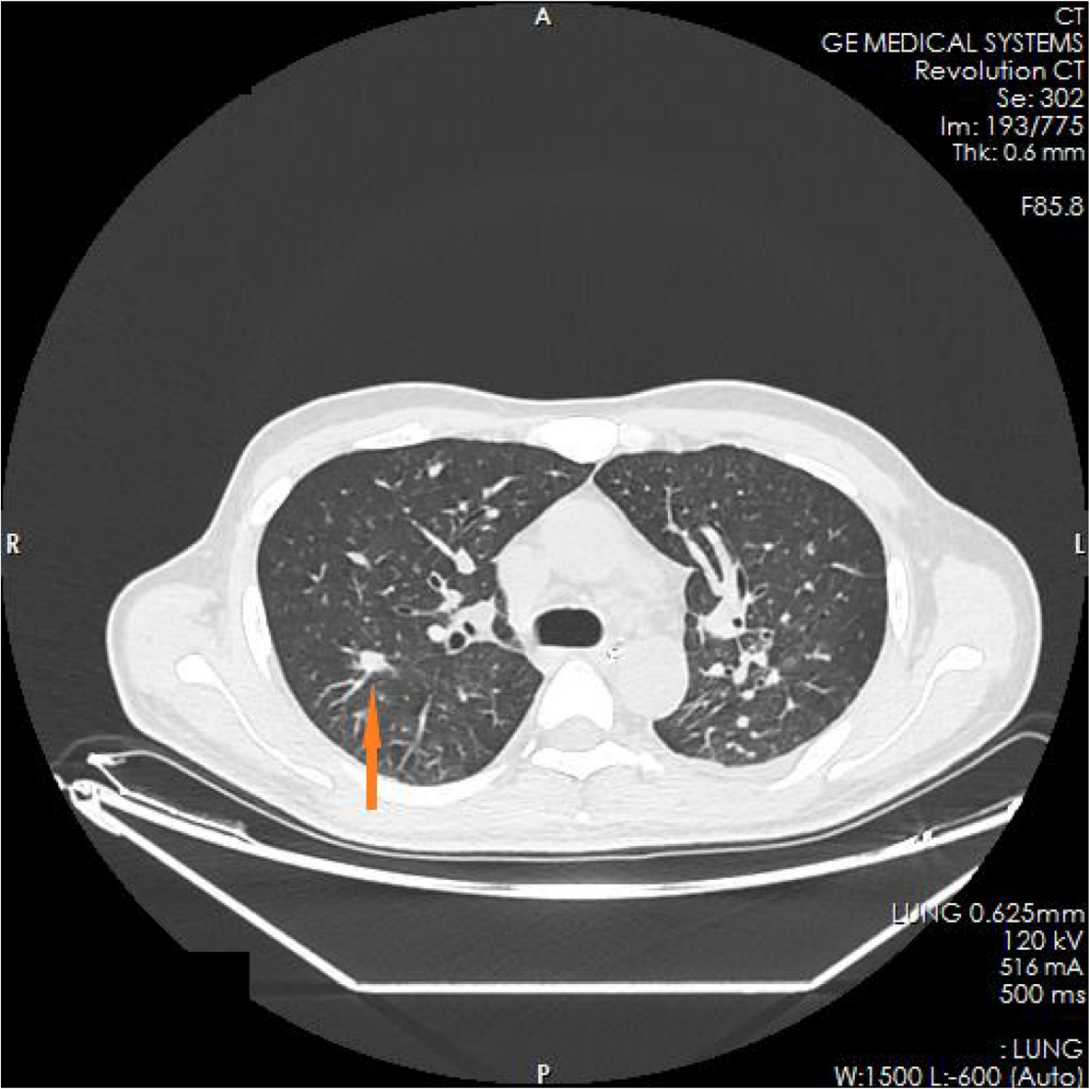
CT pictures displaying multiple tuberculosis lesions throughout the body

**Fig. 4:**
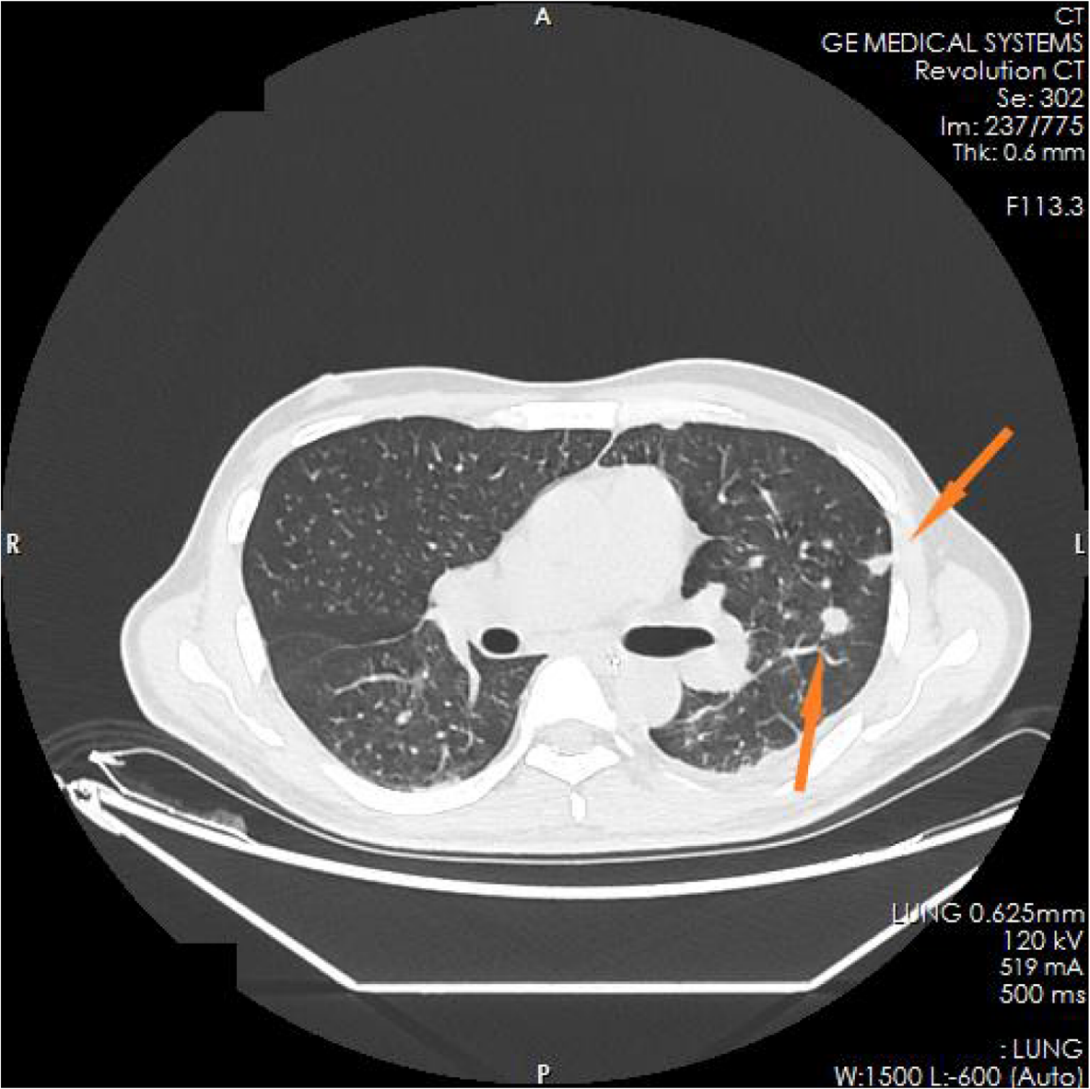
CT pictures displaying multiple tuberculosis lesions throughout the body

**Fig. 5:**
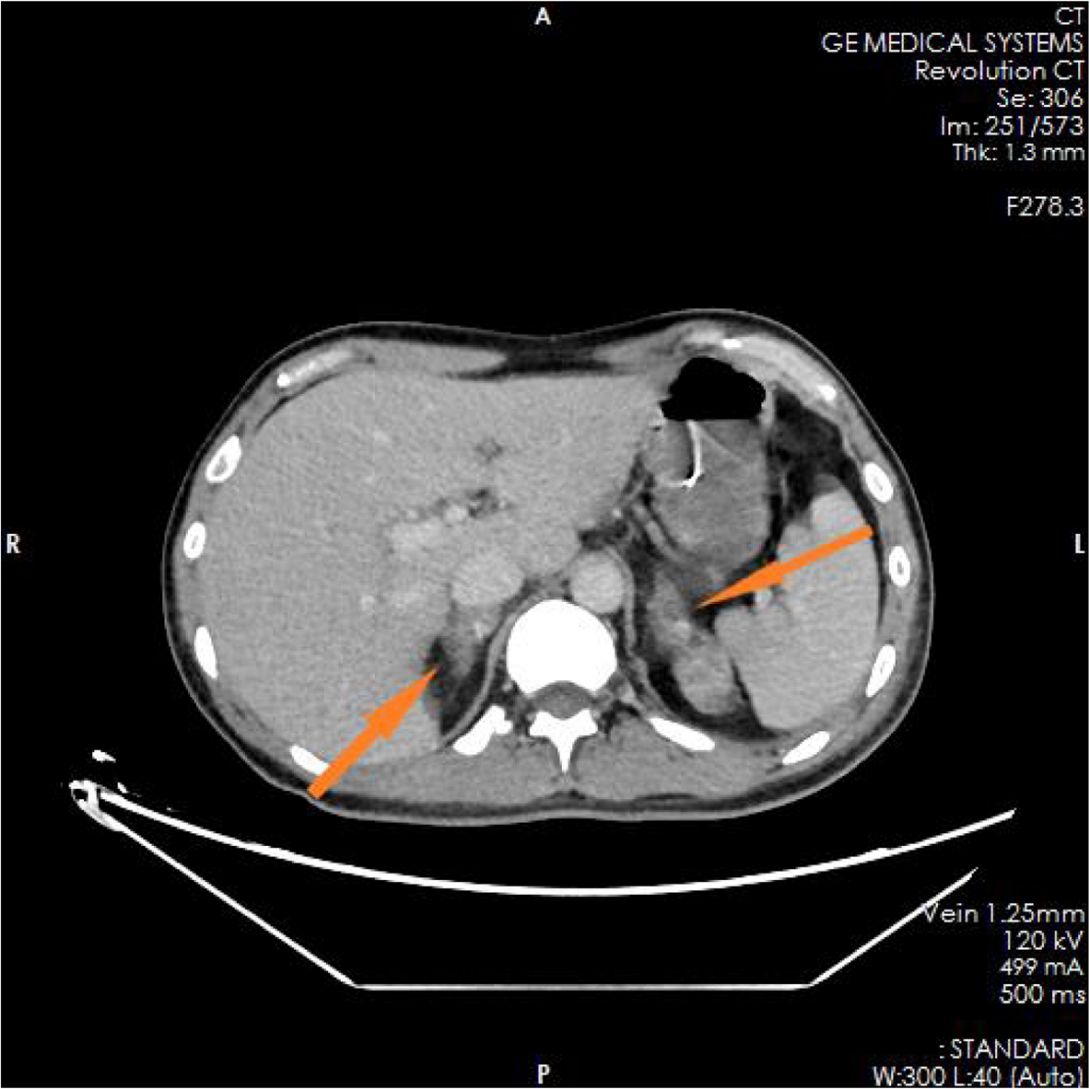
CT pictures displaying multiple tuberculosis lesions throughout the body

**Fig. 6:**
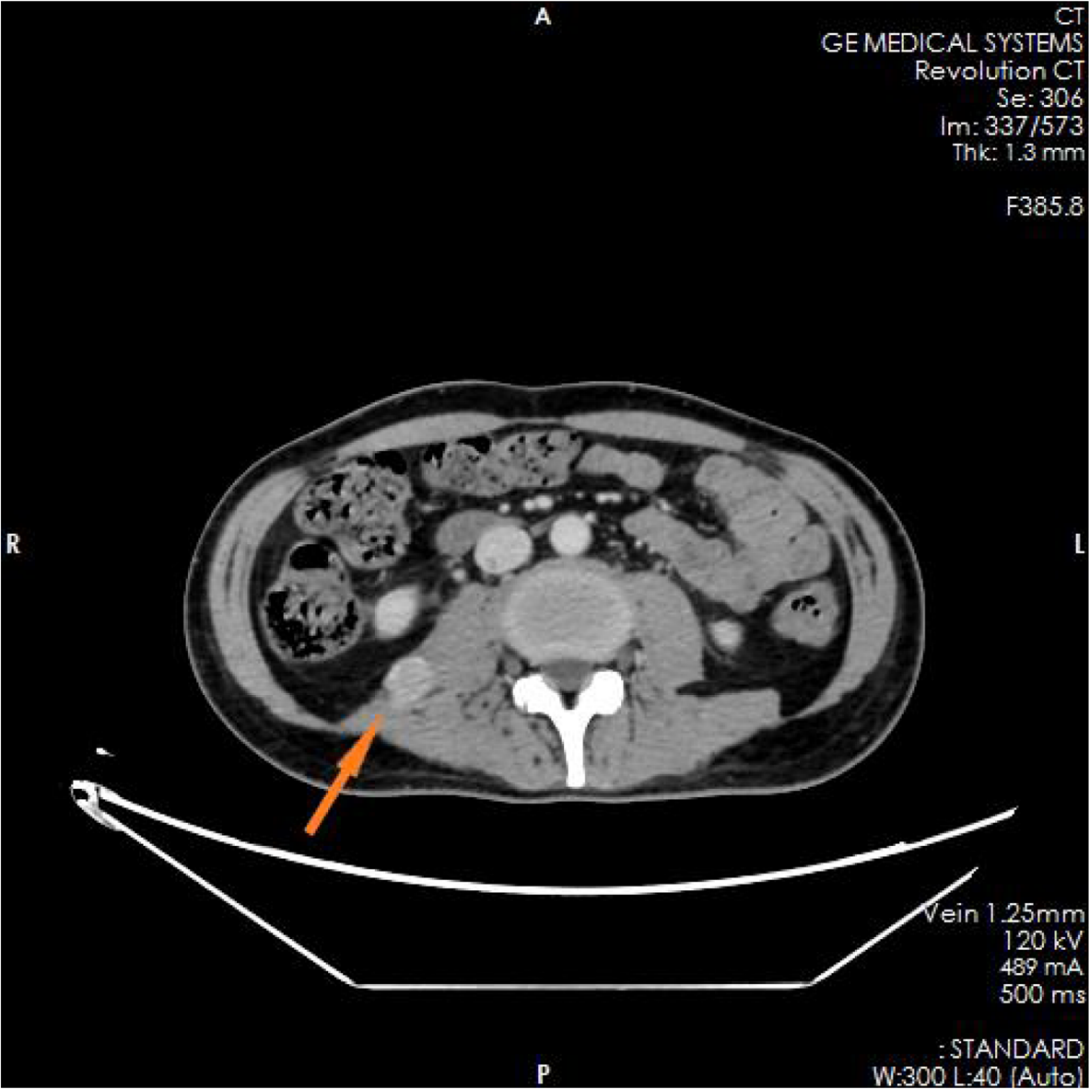
CT pictures displaying multiple tuberculosis lesions throughout the body

The family members of the patient reported that the patient had experienced nausea, vomiting, loss of appetite, and fatigue in the past 6 months. He had taken some Chinese herbs for 2 months, but his condition did not improve. He had lost approximately 10 kg in weight since the onset of illness. The test results in the ED showed blood serum potassium 6.97 mmol/l, blood serum sodium 100.9 mmol/l, and blood serum chlorine 74.1 mmol/l. Blood hormone levels during hospitalization were cortisol 2.3 μg/dl↓(8 am), 2.5 μg/dl↓(4 pm), 2.7 μg/dl↓(0 am), adrenocorticotropin (ACTH) 243.7 pmol/l↑(8 am), 270.1 pmol/l↑(4 pm), 216.2 pmol/l↑(0 am), blood aldosterone (ALD) 12 pg/ml↓, and renin (DRC) > 500 μIU/ml↑. Tuberculosis-related test results a positive *Mycobacterium tuberculosis* conformity group (MTB) DNA test, a positive rifampicin resistance (Rif Resistance) gene test, and a positive T-SPOT test (stimulation level ˃ 10, positive level 5.87, and background level 0.07). After ECMO and artificial ventilation were discontinued, imaging examination of the patient revealed multiple plaques and nodules in both lungs, multiple nodules in both adrenal glands, bone destruction of the second and third lumbar vertebrae, calcification of paravertebral soft tissue, and a mass lump in the right psoas muscle, suggesting the possibility of tuberculosis (see Figs. 1, 2, 3, 4, 5 and 6). Therefore, the patient was finally diagnosed with multiple tuberculosis infections throughout the body and adrenal tuberculosis complicated with chronic adrenal cortex insufficiency, which caused severe hyperkalemia and hyponatremia, leading to cardiac arrest.

In the EICU, the patient was given mild hypothermia, a ventilator to assist breathing, and hormone replacement therapy, to correct his electrolyte imbalance. Because the velocity time integral (VTI) of aortic flow was too low, the patient was also treated with intra-aortic balloon counterpulsation (IABP). As the blood pressure increased, we gradually reduced the dose of vasoactive drugs. After 187 h of ECMO, the heart function recovered, and the myocardial electrical activity stabilized. In the end, the patient successfully left the EICU for further rehabilitation and was transferred to the infectious disease specialist hospital for standardized anti-tuberculosis treatment.

## Discussion and conclusion

TB is one of the common infectious diseases in our country, among which lung tuberculosis is the most common, but TB bacteria can also invade many organs throughout the body, including the brain, spine, adrenal glands, and the pericardium, resulting in extrapulmonary TB. TB rarely causes acute and critical illness and usually has a chronic onset [1]. Unlike for this case, it is not common for TB patients to present to the ED with sudden cardiac arrest. Adrenal TB is one of the main causes of chronic adrenal insufficiency. The patient in this case had no typical clinical symptoms of TB, so early diagnosis and treatment were difficult. Therefore, patients with no typical TB symptoms should also be sufficiently cared for, and actively screened, and physicians should expand the scope of examination to avoid the emergence of severe conditions.

It is well known that a common cause of cardiac arrest is hyperkalemia, which itself have many causes. Primary adrenal insufficiency can cause a decrease in the secretion of aldosterone, which is a type of hormone that can simultaneously increase blood sodium levels and reduce blood potassium levels. Therefore, when the level of aldosterone is insufficient, it can cause refractory hyponatremia and hyperkalemia. Chronic adrenal insufficiency often has an insidious onset, mainly manifested by long-term poor appetite, nausea and vomiting, and fatigue [2]. These atypical symptoms make early diagnosis difficult. The patient did not have a systematic examination in time after he developed the symptoms, which eventually led to the deterioration of his condition.

ECMO is a new form of a mechanical cardiopulmonary life-support system. It involves the use of an artificial pump to transport nonoxygenated blood to a gas exchange device (oxygenator), where the blood is fully oxygenated and carbon dioxide is removed, after which the blood is reinfused back into the patient’s circulation. ECMO can partially assist the patient's cardiopulmonary function [3].

Current research indicates that the application of VA-ECMO in the ED results in good outcomes for patients with cardiac arrest requiring prolonged CPR to recover spontaneous circulation and treat refractory cardiogenic shock. ECMO plays a role as a bridge, helping provide more opportunities for the diagnosis and treatment of critically ill patients with heart and lung failure [4]. When stable hemodynamics and adequate tissue oxygen supply are ensured for the patient, physicians have more time to actively search for the cause and treat the original disease.

Therefore, ECMO can be applied in the ED to patients with cardiac arrest of different etiologies, such as acute poisoning, internal environment disturbance, and severe infection, and for patients for whom conventional CPR has difficulty stabilizing their hemodynamics. The effects of ECMO are optimal for patients between 18 and 75 years old without any irreversible end-stage conditions. Patients with reversible neurological function should be given priority to receive ECMO [5].

However, there is still much work to be done regarding the use of ECMO in the ED. High-quality prospective randomized controlled studies are needed to further evaluate the benefits and drawbacks of this procedure. More evidence-based information is needed to formulate normative guidelines. Finally, an emergency ECMO team must be established for day and night services and continuous training should be carried out for ECMO team members.

## Data Availability

All data relevant to the study are included in the article.

## Abbreviations

ECMO: Extracorporeal membrane oxygenation
ED: Emergency department
TB: Tuberculosis
CPR: Cardiopulmonary resuscitation
ACTH: Adrenocorticotropin
ALD: Aldosterone
VTI: Velocity time integral
IABP: Intra-aortic balloon counterpulsation
CA: Cardiac arrest
